# Epidemiology of transthyretin familial amyloid polyneuropathy in Portugal

**DOI:** 10.1186/1750-1172-10-S1-P21

**Published:** 2015-11-02

**Authors:** Mónica Inês, Teresa Coelho, Isabel Conceição, Filipa Duarte-Ramos, Mamede de Carvalho, João Costa

**Affiliations:** 1 Instituto de Medicina Molecular, Unidade de Fisiologia Clínica e Translacional, 1649-028, Lisboa, Portugal; 2 Centro Hospitalar do Porto, Unidade Corino de Andrade, 4000-436, Porto, Portugal; 3 Centro Hospitalar Lisboa Norte, Serviço de Neurologia, 1649-035, Lisboa, Portugal; 4 Faculdade de Farmácia da Universidade de Lisboa, Departamento de Sócio Farmácia, 1649-003, Lisboa, Portugal; 5 Instituto de Medicina Molecular, Unidade de Farmacologia Clínica, 1649-028, Lisboa, Portugal

## Background

Transthyretin Familial Amyloid Polyneuropathy (TTR-FAP) is a rare, progressive, debilitating and life-threatening neurodegenerative disease. TTR-FAP is a rare disease worldwide. In Europe a disease is defined as rare when it affects less than 1 in 2000 inhabitants. Portugal has the largest cluster worldwide nonetheless recent Portuguese epidemiologic data is lacking. The purpose of this study is to estimate TTR-FAP prevalence in Portugal.

## Methods

In Portugal, TTR-FAP patient's medicines utilization is fully funded by National Health Service since 2001. Since March 2013 Portuguese electronic prescription system became more generalized, allowing central monitoring and validation of medicines prescription and dispensing. TTR-FAP anonymized patient's prescription data was requested to Administração Central do Sistema Saúde (ACSS). For each prescription the database has information regarding the local where the medicines were dispensed. Hence, for each patient, the most frequent municipality was identified and used as a proxy for residence. Portuguese total population by municipality was obtained from the official source - Instituto Nacional de Estatística. Prevalence was reported for mainland country and by municipality as number of cases per 2000 inhabitants.

## Results

Trough year 2014, a total of 70286 electronic dispensing acts were detected in ACSS database, unravelling a total of 2013 distinctive TTR-FAP patients in Portugal. A prevalence of 0,41 per 2000 inhabitants was estimated for TTR-FAP in Portugal. The disease is currently spread across 160 of the 278 Portuguese municipalities and in 19 of them affects more than 1 per 2000 inhabitants. The municipalities with higher TTR-FAP prevalence are: Póvoa de Varzim, Pampilhosa da Serra, Seia, Esposende, Vila do Conde, Figueira da Foz, Boticas and Barcelos.

## Conclusion

We can estimate that TTR-FAP disease has a current prevalence of 0,41 per 2000 inhabitants in Portugal and it is now disseminated across the country being Póvoa de Varzim historically and still the most impacted municipality with 3,95 cases per 2000 inhabitants, nearly ten times higher than overall country prevalence.

